# Idelalisib exposure before allogeneic stem cell transplantation in patients with follicular lymphoma: an EBMT survey

**DOI:** 10.1038/s41409-020-0946-x

**Published:** 2020-05-22

**Authors:** Leopold Sellner, Johannes Schetelig, Linda Koster, Goda Choi, Didier Blaise, Dietrich Beelen, Fabrizio Carnevale Schianca, Jakob Passweg, Urs Schanz, Emmanuel Gyan, Federica Sora, Nicolaus Kröger, Gerald. G. Wulf, Gwendolyn Van Gorkom, Jiri Mayer, Corentin Orvain, Jean Henri Bourhis, Pavel Jindra, Victoria Potter, Francesco Zallio, Elisabeth Vandenberghe, Stephen Robinson, Patrick J. Hayden, Ibrahim Yakoub-Agha, Silvia Montoto, Peter Dreger, on behalf of the European Society for Blood and Marrow Transplantation Lymphoma and Chronic Malignancies Working Parties

**Affiliations:** 1 University Hospital Heidelberg, Heidelberg, Germany; 2 University of Dresden, Dresden, Germany; 3 EBMT Data Office, Leiden, The Netherlands; 4 University Medical Center Groningen, University of Groningen, Groningen, The Netherlands; 5 Aix-Marseille Univ, Inserm, CNRS, Institut Paoli-Calmettes, CRCM, Marseille, France; 6 University Hospital of Essen, Essen, Germany; 7 Ospedale Infantile Regina Margherita, Turin, Italy; 8 University Hospital of Basel, Basel, Switzerland; 9 University Hospital of Zurich, Zurich, Switzerland; 10 Hematology and cell therapy department, Centre hospitalier Universitaire, Université de Tours, CIC Inserm 1415, Tours, France; 11 Sez. Ematologia, Fondazione Policlinico Universitario A. Gemelli—IRCCS, Università Cattolica Sacro Cuore, Rome, Italy; 12 University Hospital Eppendorf, Hamburg, Germany; 13 University Hospital Goettingen, Goettingen, Germany; 14 University Hospital Maastricht, Maastricht, The Netherlands; 15 University Hospital Brno, Brno, Czech Republic; 16 University Hospital of Angers, Angers, France; 17 Gustave Roussy Cancer Campus, Villejuif, France; 18 Charles University Hospital, Pilsen, Czech Republic; 19 GKT School of Medicine, London, UK; 20 Divisione di Ematologia, AO SS Antonio e Biagio e Cesare Arrigo Alessandria, Alessandria, Italy; 21 Department of Haematology, Trinity College Dublin, St. James’s Hospital, Dublin 8, Ireland; 22 Bristol Royal Hospital for Children, Bristol, UK; 23 Univ. Lille, Inserm, CHU Lille, INSERM, Infinite, U1286F-59000, Lille, France; 24 St. Bartholomew’s Hospital, Barts Health NHS Trust, London, UK; 25 Present Address: Takeda Pharma Vertrieb GmbH & Co. KG, Berlin, Germany

**Keywords:** Targeted therapies, Clinical trials, Cancer immunotherapy

## To the Editor:

The PI3Kδ inhibitor idelalisib is approved for the treatment of relapsed or refractory (R/R) follicular lymphoma (FL) [1, 2]. However, the duration of response is mostly limited, especially in patients who do not achieve a complete response (CR). A median progression-free survival (PFS) of only 11 months was reported in patients in partial remission (PR) compared with 30.6 months in patients in CR [2]. Therefore, effective consolidation will be necessary to achieve long-term remissions. Allogeneic stem cell transplantation (alloSCT) is an option in patients responding to idelalisib [3, 4], though there is limited information on the impact of previous exposure to idelalisib on the feasibility and safety of alloSCT. This is of particular importance as immune-mediated toxicities of idelalisib including hepatitis, colitis, pneumonitis, and skin rash, probably mediated by selective inhibition of regulatory T cells, may interfere with a subsequent allogeneic stem cell transplant [5, 6].

The aim of this European Society for Blood and Marrow Transplantation (EBMT) registry study (study code LWP 2013-N-03) was to assess the safety and efficacy of alloSCT after prior exposure to idelalisib in patients with FL. Patients aged ≥ 18 years who underwent a first alloSCT for FL after exposure to idelalisib at any time before transplant between 2015 and 2018 and who were registered with the EBMT were eligible for inclusion. Any donor type and any conditioning regimen were allowed. Baseline patient, disease, and transplant data were collected from MED-A forms. Centers were requested to provide additional treatment and follow-up information (MED-B and C forms). The primary endpoint was nonrelapse mortality (NRM) at 6 and 12 months post alloSCT. Secondary endpoints were overall survival (OS) and PFS as well as incidence of relapse (RI), engraftment, and acute or chronic graft-versus-host disease (GvHD). Informed consent for transplantation and data collection was obtained locally according to the regulations applicable at the time of transplantation. All transplant centers have been required to obtain written informed consent prior to data registration with the EBMT following the 1964 Helsinki declaration and its later amendments. Statistical analysis was performed using the survival and cmprsk packages in R 3.3.2 (R Foundation for Statistical Computing, Vienna, Austria, http://www.R-project.org). Survival curves were estimated by the Kaplan–Meier method. Cumulative incidence taking into account competing risks were estimated for NRM and RI.

Sixty-three patients met the eligibility criteria. Eighteen were excluded because of missing follow-up, unclear idelalisib starting date, or unclear exposure to idelalisib, leaving 45 patients for analysis. A total of 33 of these patients received idelalisib for bridging to alloSCT (as last line before transplant). Overall, 80% of all patients were in PR or better at alloSCT, including 20% CRs. In patients who had received idelalisib for bridging to alloSCT, 82% had responded to the idelalisib-containing regimen with 15% of the patients in CR. The median follow-up was 12 months. Patient characteristics details are shown in Table 1.

**Table 1:** Patient characteristics.

Patients with follicular lymphoma	All (*n* = 45)
Age at alloSCT, median (range), years	57 (34–71)
Female gender, *n* (%)	18 (40)
Pretreatments before alloSCT, median (range)	4 (2–8)
Remission status at alloSCT, *n* (%)	
CR	9 (20)
PR	27 (60)
SD	1 (2)
PD/primary refractory	6 (13)
Unknown	2 (4)
Good performance status at alloSCT (Karnofsky 90–100%), *n* (%)	30/42 (71)
Matched related donor *n* (%)	12 (27)
Conditioning, *n* (%)	
TBI based	13 (29)
Alkylator based	32 (71)
Alemtuzumab	5 (11)
ATG	22 (49)
Reduced intensity conditioning^a^ (RIC), *n* (%)	31 (69)
Idelalisib administration, *n* (%)	
Monotherapy	
Anti-CD20 antibody combination	11 (24)
Chemotherapy combination	2 (4)
AutoSCT prior to alloSCT, *n* (%)	22 (49)
Median follow-up of survivors, months after alloSCT (range)	12 (2–35)

*alloSCT* allogeneic stem cell transplantation, *ATG* anti-thymoglobulin, *autoSCT* autologous stem cell transplantation, *CR* complete remission, *PD* progressive disease, *PR* partial remission, *SD* stable disease, *TBI* total body irradiation.

^a^According to the EBMT criteria.

**Fig. 1:**
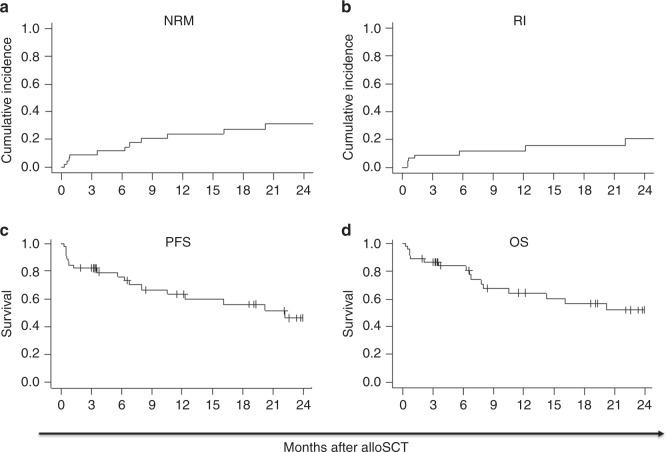
Outcome of follicular lymphoma patients after allogeneic stem cell transplantation. Nonrelapse mortality (NRM; **a**), incidence of relapse (RI; **b**), progression-free survival (PFS; **c**), and overall survival (OS; **d**) for patients with idelalisib exposure before allogeneic stem cell transplantation (alloSCT).

The median time to reach neutrophils > 0.5/nl and platelets > 20/nl was 16 days post transplant. Two patients failed to engraft, both due to early death from infection, 10 and 23 days post alloSCT, respectively. Acute GVHD (aGvHD) grade 2–4 was observed in 45% and grade 3–4 in 24% of patients. Overall, 35% of evaluable patients-at-risk developed chronic GvHD (cGvHD), and 10% were classified as extensive disease. Eleven NRM events were reported, four within the first month, two at 6 months, and one each at 3, 7, 10, 16, and 20 months post alloSCT. Causes of death included GvHD (*n* = 6), infection (*n* = 5), relapse (*n* = 2), GI toxicity (*n* = 1), and secondary malignancy (*n* = 1). Six- and twelve-month incidences of NRM were 12 and 24% (Fig. 1a), RI were 12 and 12% (Fig. 1b), PFS were 76 and 64% (Fig. 1c), and OS were 84 and 64% (Fig. 1d). Disease status at transplant (SD/PR versus CR) did not have an impact on outcome in the current analysis. Outcomes of patients who received idelalisib directly before alloSCT for bridging were comparable with the entire cohort.

The outcome observed in this series seems to be comparable with that reported in the largest study so far on alloSCT in FL, where 1567 patients from the Center for International Blood and Marrow Transplant Research and the EBMT databases who were not previously exposed to idelalisib were retrospectively analyzed with 3-year NRM, RI, PFS, and OS estimates of 25, 17, 58, and 66% (95% confidence interval 23–27, 15–19, 55–60, and 64–68%) [4]. With a shorter follow-up in the current study, 12-month outcomes are in line with the findings reported by Sureda et al. [4] Although grade 2–4 aGvHD in nearly half of our cohort (45%) seems to be higher compared with the study by Sureda et al. that reported 20% aGvHD grade 2–4 [4], it is in keeping with other registry studies on alloSCT in FL [7, 8]. In contrast, there were no signs of an increased incidence of cGvHD after pretransplant idelalisib exposure.

While long-term results of CAR trials are still awaited, alloSCT remains the only potentially curative treatment option in advanced stage FL, even though late relapses can occur [4, 9–11]. AutoSCT may also provide long-term remissions in chemo-sensitive disease but a significant proportion of patients will eventually relapse [12]. Other specific pathway inhibitors such as the BTK inhibitor ibrutinib [13] or the BCL-2 inhibitor venetoclax [14] may be additional options in R/R FL but are not approved for this indication. Our results suggest that idelalisib does not increase the risk of NRM or GVHD when used pre transplant. This has been a concern since idelalisib has been shown to suppress the function of regulatory T cells, which may increase the risk of immunologic complications post alloSCT [15].

In summary, the outcome of patients who received idelalisib before alloSCT was comparable with previous reports of alloSCT in FL patients without idelalisib pretreatment. Idelalisib seems to be an effective and safe drug for bridging patients with FL to alloSCT, especially in the chemotherapy-refractory setting, with a high percentage of patients with responding disease at the time of transplant. However, further studies are required to confirm that idelalisib bridging does not increase the risk of aGvHD.
