# Correction: Idelalisib exposure before allogeneic stem cell transplantation in patients with follicular lymphoma: an EBMT survey

**DOI:** 10.1038/s41409-021-01490-9

**Published:** 2021-10-07

**Authors:** Leopold Sellner, Johannes Schetelig, Linda Koster, Goda Choi, Didier Blaise, Dietrich Beelen, Fabrizio Carnevale Schianca, Jakob Passweg, Urs Schanz, Emmanuel Gyan, Federica Sora, Nicolaus Kröger, Gerald. G. Wulf, Gwendolyn Van Gorkom, Jiri Mayer, Corentin Orvain, Jean Henri Bourhis, Pavel Jindra, Victoria Potter, Francesco Zallio, Elisabeth Vandenberghe, Stephen Robinson, Patrick J. Hayden, Ibrahim Yakoub-Agha, Silvia Montoto, Peter Dreger, on behalf of the European Society for Blood and Marrow Transplantation Lymphoma and Chronic Malignancies Working Parties

**Affiliations:** 1 University Hospital Heidelberg, Heidelberg, Germany; 2 University of Dresden, Dresden, Germany; 3 EBMT Data Office, Leiden, The Netherlands; 4 University Medical Center Groningen, University of Groningen, Groningen, The Netherlands; 5 Aix-Marseille Univ, Inserm, CNRS, Institut Paoli-Calmettes, CRCM, Marseille, France; 6 University Hospital of Essen, Essen, Germany; 7 Ospedale Infantile Regina Margherita, Turin, Italy; 8 University Hospital of Basel, Basel, Switzerland; 9 University Hospital of Zurich, Zurich, Switzerland; 10 Hematology and cell therapy department, Centre hospitalier Universitaire, Université de Tours, CIC Inserm 1415, Tours, France; 11 Sez. Ematologia, Fondazione Policlinico Universitario A. Gemelli—IRCCS, Università Cattolica Sacro Cuore, Rome, Italy; 12 University Hospital Eppendorf, Hamburg, Germany; 13 University Hospital Goettingen, Goettingen, Germany; 14 University Hospital Maastricht, Maastricht, The Netherlands; 15 University Hospital Brno, Brno, Czech Republic; 16 University Hospital of Angers, Angers, France; 17 Gustave Roussy Cancer Campus, Villejuif, France; 18 Charles University Hospital, Pilsen, Czech Republic; 19 GKT School of Medicine, London, UK; 20 Divisione di Ematologia, AO SS Antonio e Biagio e Cesare Arrigo Alessandria, Alessandria, Italy; 21 Department of Haematology, Trinity College Dublin, St. James’s Hospital, Dublin 8, Ireland; 22 Bristol Royal Hospital for Children, Bristol, UK; 23 Univ. Lille, Inserm, CHU Lille, INSERM, Infinite, U1286F-59000, Lille, France; 24 St. Bartholomew’s Hospital, Barts Health NHS Trust, London, UK; 25 Present Address: Takeda Pharma Vertrieb GmbH & Co. KG, Berlin, Germany

**Keywords:** Targeted therapies, Clinical trials, Cancer immunotherapy

Correction to: *Bone Marrow Transplantation* 10.1038/s41409-020-0946-x, published online 22 May 2020

The article “Idelalisib exposure before allogeneic stem cell transplantation in patients with follicular lymphoma: an EBMT survey”, written by Leopold Sellner, Johannes Schetelig, Linda Koster, Goda Choi, Didier Blaise, Dietrich Beelen, Fabrizio Carnevale Schianca, Jakob Passweg, Urs Schanz, Emmanuel Gyan, Federica Sora, Nicolaus Kröger, Gerald. G. Wulf, Gwendolyn Van Gorkom, Jiri Mayer, Corentin Orvain, Jean Henri Bourhis, Pavel Jindra, Victoria Potter, Francesco Zallio, Elisabeth Vandenberghe, Stephen Robinson, Patrick J. Hayden, Ibrahim Yakoub-Agha, Silvia Montoto, Peter Dreger, on behalf of the European Society for Blood and Marrow Transplantation Lymphoma and Chronic Malignancies Working Parties, was originally published Online First without Open Access. After publication in volume 55, issue 12, page 2335–2338, the author decided to opt for Open Choice and to make the article an Open Access publication. Therefore, the copyright of the article has been changed to © The Author(s) 2020 and the article is forthwith distributed under the terms of the Creative Commons Attribution 4.0 International License, which permits use, sharing, adaptation, distribution and reproduction in any medium or format, as long as you give appropriate credit to the original author(s) and the source, provide a link to the Creative Commons licence, and indicate if changes were made. The images or other third party material in this article are included in the article’s Creative Commons licence, unless indicated otherwise in a credit line to the material. If material is not included in the article’s Creative Commons licence and your intended use is not permitted by statutory regulation or exceeds the permitted use, you will need to obtain permission directly from the copyright holder. To view a copy of this licence, visit http://creativecommons.org/licenses/by/4.0. Open access funding is enabled and organized by Projekt DEAL.

